# Supplementary material for: Multilocus Sex Determination Revealed in Two Populations of Gynodioecious Wild Strawberry, Fragaria vesca subsp. bracteata
Source: G3 (Bethesda). 2015 Oct 16;5(12):2759–73. doi: 10.1534/g3.115.023358 (PMC4683647; doi:10.1534/g3.115.023358)
Supplement: Supporting Information [file supp_5_12_2759__index.html]

Multilocus Sex Determination Revealed in Two Populations of Gynodioecious Wild Strawberry, Fragaria vesca subsp. bracteata — Supporting Information 

# Multilocus Sex Determination Revealed in Two Populations of Gynodioecious Wild Strawberry, *Fragaria vesca* subsp. *bracteata*

## Supporting Information for Ashman *et al.*, 2015

**Files in this Data Supplement:**

- Table S1 - Cytotypes of *F. vesca* subsp. *bracteata* parents used in the crossing study. (.docx, 17 KB)
- Table S2 - Primer sequences (forward/reverse), annealing temperatures and reference genome coordinates for eight informative polymorphic sites segregating with male sterility identified OR-MRD30xOR-MRD60 and NM-LNF23 self-map cross population. (.docx, 16 KB)
- Table S3 - Genomic locations, functional annotations, and PLAZA 3.0 gene families of genes at the Fvb6 male sterility locus in *Fragaria vesca* subsp. *bracteata*. (.docx, 95 KB)
- Table S4 - Genomic locations and PLAZA 3.0 gene families of PPR genes at the peach RF1 and RF2 loci (Donoso et al. 2015). (.docx, 19 KB)
