# Supplementary material for: Multilocus Sex Determination Revealed in Two Populations of Gynodioecious Wild Strawberry, Fragaria vesca subsp. bracteata
Source: G3 (Bethesda). 2015 Oct 16;5(12):2759–73. doi: 10.1534/g3.115.023358 (PMC4683647; doi:10.1534/g3.115.023358)
Supplement: Supporting Information [file supp_g3.115.023358_TableS1.docx]

Table S1. Cytotypes of *F. vesca* subsp. *bracteata* parents used in the crossing study. Population and plant ids and sex types are given along with sequence for chloroplast and mitochondrial genes. Cytotype codes follow Stanley et al. (2015).

|  |  |  | **Cytotypes** | | | |
| --- | --- | --- | --- | --- | --- | --- |
|  |  |  | **Chroloplast** | **Mitochondria** | | |
| **Population** | **Plant ID** | **Sex** | *rpoC2* (code) | *atp8* | *atp8-orf225* | code |
| OR-MRD | OR-MRD93 | H | *C* (2) | *GAGGC* | *TAG* | B |
|  | OR-MRD61 | H | *C* (2) | *GAGGC* | *TCG* | C |
|  | OR-MRD45 | H | *C* (2) | *GAGGC* | *TCG* | C |
|  | OR-MRD30 | F | *C* (2) | *GAGGC* | *TCG* | C |
|  | OR-MRD27 | F | *C* (2) | *GAGGC* | *TCG* | C |
|  | OR-MRD90 | F | *C* (2) | *GAGGC* | *TCG* | C |
| NM-LNF | NM-LNF23 | H | *G* (1) | *GAGTC* | *GCG* | F |
|  | NM-LNF25 | H | *G* (1) | *GAGTC* | *GCG* | F |
|  | NM-LNF14 | H | *G* (1) | *GAGTC* | *GCG* | F |
|  | NM-LNF2 | F | *G* (1) | *GAGTC* | *GCG* | F |
|  | NM-LNF4 | F | *G* (1) | *GAGTC* | *GCG* | F |
|  | NM-LNF26 | F | *G* (1) | *GAGTC* | *GCG* | F |
