# Supplementary material for: Multilocus Sex Determination Revealed in Two Populations of Gynodioecious Wild Strawberry, Fragaria vesca subsp. bracteata
Source: G3 (Bethesda). 2015 Oct 16;5(12):2759–73. doi: 10.1534/g3.115.023358 (PMC4683647; doi:10.1534/g3.115.023358)
Supplement: Supporting Information [file supp_g3.115.023358_TableS2.docx]

Table S2   Primer sequences (forward/reverse), annealing temperatures and reference genome coordinates for eight informative polymorphic sites segregating with male sterility identified OR-MRD30xOR-MRD60 and NM-LNF23 self-map cross population. Coordinates in FvH4 are with respect to the *F. vesca* ssp. *vesca* reference genome version 1. 0 (Shulev et al. 2010), while those in bold are on the scaffold scf0513158b (Tennessen et al. 2013). Coordinates in Fvb are with respect to *F. vesca* subsp. *bracteata* assembly of the reference genome version 1.0 (‘Fvb’ described in Tennessen et al. 2014).

| **Linkage group** | **Locus** | **Coordinates in FvH4** | **Coordinates on Fvb** | **Forward/ Reverse**  **primer sequences (5' - 3')** | **Annealing temperature °C - Time** |
| --- | --- | --- | --- | --- | --- |
| LG4 | scB_1731089 | **1730768-1730793** | Fvb4_30092397-30092422 | For: GCCCTGTTGTAGAAATGAATCTGGAG |  |
|  | Fvb4_30092k | **1731360-1731383** | Fvb4_30092989-30093012 | Rev: TTATTCCCCCTACTCACACTCAGC | 56°C - 30 sec |
| Fvb-s 6 | Fvb-s 6_33035k | 33035707-33035731 | Fvb6_34763127-34763151 | For: CCAACTCCAAGGAAGAGGTTTTGTG |  |
|  | Fvb6_34763k | 33036405-33036431 | Fvb6_34763825-34763851 | Rev: ACAGGCTTCATGTAATTCAGATTTGTC | 55°C - 30 sec |
| Fvb-s 6 | Fvb-s 6_33414k | 33414605-33414630 | Fvb6_35142025-35142050 | For: CCATTCCTTTTGGTTGCTTTCATGC |  |
|  | Fvb6_35142k | 33415104-33415129 | Fvb6_35142524-35142549 | Rev: TTTCATCTGGTTTTCCCTTGGTTGG | 55°C - 30 sec |
| Fvb-s 6 | Fvb-s 6_36455k | 36455635-36455659 | Fvb6_36607045-36607069 | For: TTTTGACAGCCCTACGTTCATCAAG |  |
|  | Fvb6_36607k | 36455972-36455997 | Fvb6_36607382-36607407 | Rev: CAGCCACAAAGAAAACAAATTCAGGT | 55°C - 30 sec |
