# Supplementary material for: Multilocus Sex Determination Revealed in Two Populations of Gynodioecious Wild Strawberry, Fragaria vesca subsp. bracteata
Source: G3 (Bethesda). 2015 Oct 16;5(12):2759–73. doi: 10.1534/g3.115.023358 (PMC4683647; doi:10.1534/g3.115.023358)
Supplement: Supporting Information [file supp_g3.115.023358_TableS3.docx]

| Table S3.  Genomic locations, functional annotations, and PLAZA 3.0 gene families of genes at the Fvb6 male sterility locus in *Fragaria vesca* subsp. *bracteata*. Notes: 1: Pentatricopeptide repeats (PPR), 2: the PPR gene family that contains known and suspected fertility restorers, 3: F-box proteins observed to be upregulated in meiotic anthers at stage 9, 4: miRNA target (Xia et al 2015), 5: unique to Darwish et al. 2015 annotation. | | | | | | | |
| --- | --- | --- | --- | --- | --- | --- | --- |
| **Gene** | **Fvb6 start** | **Fvb6 end** | **PLAZA 3.0 gene family** | **PLAZA 3.0 orthology group** | **PLAZA 3.0 annotation (Proost et al 2015)** | **Gene expression (Hollander et al 2014)** | **Notes** |
| gene04313 | 34839670 | 34840649 | HOM03D006135 | ORTHO03D181430 | Putative F-box protein At5g14160 (probable) |  |  |
| gene04314 | 34842621 | 34843766 | HOM03D006135 | ORTHO03D028177 | F-box protein At2g05970 (probable) | upregulated anther stage 11 vs. anther stage 10 |  |
| gene34746 | 34845812 | 34846894 | HOM03D000822 | ORTHO03D000160 | Translation initiation factor IF-2 (probable) |  |  |
| gene34747 | 34847317 | 34848872 | HOM03D000822 | ORTHO03D000160 | Ribosome-binding protein 1 (mRRp) (probable) |  |  |
| gene34748 | 34849305 | 34857180 | HOM03D000822 | ORTHO03D000160 | Ribosome-binding protein 1 (mRRp) (probable) |  |  |
| gene04315 | 34858207 | 34859526 | HOM03D006135 | ORTHO03D028177 | Putative F-box protein At3g25750 (probable) |  |  |
| gene04316 | 34875629 | 34882912 | HOM03D000948 | ORTHO03D001391 | U5 small nuclear ribonucleoprotein 200 kDa helicase (U5-200KD) (similar to) | male gametophyte specific, upregulated pollen vs. anther stage 12, upregulated microspore vs. anther stage 10 |  |
| gene04317 | 34883190 | 34884425 | HOM03D003217 | ORTHO03D197748 | Putative FBD-associated F-box protein At5g22720 (probable) | upregulated anther stage 9 vs. anther stage 7-8, upregulated anther stage 9 vs. anther stage 10 | 3 |
| gene04318 | 34886277 | 34887896 | HOM03D000740 | ORTHO03D014945 | Histidine-rich glycoprotein, Precursor (probable) |  |  |
| gene04319 | 34888607 | 34889705 | HOM03D002274 | ORTHO03D256614 | Glycyl-tRNA synthetase 1, mitochondrial (GlyRS 1), Precursor (probable) |  |  |
| gene04320 | 34890053 | 34891660 | HOM03D001429 | ORTHO03D013766 | Zinc finger protein 569 (Zfp-74) (probable),PlantTFDB=C2H2 | upregulated anther stage 11 vs. anther stage 10 |  |
| gene04321 | 34893730 | 34895890 | HOM03D000048 | ORTHO03D045002 | fatm_receptor-like protein kinase At1g67000 |  |  |
| gene04322 | 34896894 | 34903489 | HOM03D001270 | ORTHO03D005085 |  | upregulated anther stage 10 vs. microspore |  |
| gene04323 | 34906094 | 34907612 | HOM03D005623 | ORTHO03D009148 | fatm_chaperone regulator 8, chloroplastic | upregulated anther stage 12 vs. pollen, upregulated microspore vs. anther stage 10 |  |
| gene04324 | 34908588 | 34911401 | HOM03D004830 | ORTHO03D003467 | DNA-directed RNA polymerase I subunit rpa49 (RNA polymerase I subunit A49) (probable) |  |  |
| gene04325 | 34911791 | 34912444 | HOM03D002117 | ORTHO03D274402 | 25.3 kDa vesicle transport protein (AtSEC22) (putative) | upregulated anther stage 9 vs. anther stage 7-8, upregulated anther stage 9 vs. anther stage 10 |  |
| gene04326 | 34916509 | 34917534 | HOM03D000321 | ORTHO03D005344 | tRNA (adenine-N(1)-)-methyltransferase non-catalytic subunit TRM6 (tRNA(m1A58)MTase subunit TRM6) (probable) | upregulated anther stage 7-8 vs. anther stage 9, upregulated anther stage 11 vs. anther stage 12 |  |
| gene04327 | 34918514 | 34919307 | HOM03D002849 | ORTHO03D318929 | Tyrosyl-tRNA synthetase (TyrRS) (similar to) | upregulated anther stage 12 vs. anther stage 11 |  |
| gene04328 | 34919335 | 34920911 | HOM03D002849 | ORTHO03D325684 | Tyrosyl-tRNA synthetase (TyrRS) (probable) |  |  |
| gene04329 | 34921498 | 34922204 | HOM03D003439 | ORTHO03D002063 | LYR motif-containing protein 4 (probable) | upregulated anther stage 10 vs. microspore |  |
| gene04330 | 34922692 | 34924088 | HOM03D000001 | ORTHO03D008702 | Pentatricopeptide repeat-containing protein At5g39350 (similar to) | upregulated microspore vs. anther stage 10 | 1 |
| gene04331 | 34925225 | 34927425 | HOM03D001224 | ORTHO03D121804 | Putative nucleosome assembly protein C364.06 (probable) | upregulated anther stage 12 vs. anther stage 11 |  |
| gene04332 | 34928234 | 34928786 | HOM03D000831 | ORTHO03D298171 | Lateral signaling target protein 2 homolog (probable) | upregulated pollen vs. anther stage 12 |  |
| gene04333 | 34933770 | 34938063 | HOM03D000188 | ORTHO03D001147 | Glycosyltransferase QUASIMODO1 (probable) | upregulated anther stage 12 vs. anther stage 11, upregulated pollen vs. anther stage 12 |  |
| gene04334 | 34939438 | 34942509 | HOM03D000804 | ORTHO03D012811 | Membrane-anchored ubiquitin-fold protein 1 (Membrane-anchored ub-fold protein 1), Precursor (similar to) |  |  |
| gene04335 | 34944106 | 34945638 | HOM03D001396 | ORTHO03D001812 | Putative prolyl-tRNA synthetase C19C7.06 (ProRS) (probable) | upregulated anther stage 12 vs. anther stage 11 |  |
| gene04336 | 34946801 | 34947967 | HOM03D000018 | ORTHO03D344062 | F-box/kelch-repeat protein At3g06240 (probable) | upregulated anther stage 9 vs. anther stage 7-8, upregulated anther stage 9 vs. anther stage 10 | 3 |
| gene04337 | 34949210 | 34954623 | HOM03D000388 | ORTHO03D000352 | Chaperone protein clpB 2 (similar to) | upregulated anther stage 12 vs. pollen |  |
| gene04338 | 34955703 | 34956626 | HOM03D006845 | ORTHO03D008972 | Putative amino acid permease F13H10.3 (probable) |  |  |
| gene04339 | 34958505 | 34959038 | HOM03D002338 | ORTHO03D005699 | Phytochrome A-associated F-box protein (probable) |  |  |
| gene04340 | 34961992 | 34963107 | HOM03D002353 | ORTHO03D028626 | F-box protein At4g19940 (probable) | upregulated anther stage 10 vs. microspore |  |
| gene34749 | 34965172 | 34967280 | HOM03D000134 | ORTHO03D014809 | RAF proto-oncogene serine/threonine-protein kinase (probable) |  |  |
| gene04341 | 34969216 | 34969617 | HOM03D002252 | ORTHO03D049470 | Translation initiation factor IF-2 (probable) |  |  |
| gene04342 | 34972856 | 34973851 | HOM03D001455 | ORTHO03D002522 | Putative HTH-type transcriptional regulator ykgA (probable) |  |  |
| gene04343 | 34974570 | 34975063 | HOM03D002353 | ORTHO03D028626 | Protein strawberry notch (probable) |  |  |
| gene04344 | 34981405 | 34982544 | HOM03D002353 | ORTHO03D028626 | F-box/kelch-repeat protein At3g06240 (probable) | upregulated anther stage 9 vs. anther stage 7-8 | 3 |
| gene04345 | 34982992 | 34984806 | HOM03D002730 | ORTHO03D009437 | Myb-like protein V (probable) | upregulated anther stage 12 vs. pollen, upregulated microspore vs. anther stage 10 |  |
| gene04346 | 34989170 | 34991549 | HOM03D005365 | ORTHO03D006675 | Homoserine O-acetyltransferase (Homoserine transacetylase) (probable) | upregulated anther stage 10 vs. microspore |  |
| gene04347 | 34992341 | 34993141 | HOM03D002120 | ORTHO03D003308 | RING finger protein 208 (probable) | upregulated pollen vs. anther stage 12, upregulated anther stage 10 vs. microspore |  |
| gene04348 | 35001105 | 35004797 | HOM03D000120 | ORTHO03D000486 | Cyclic nucleotide-gated ion channel 2 (AtCNGC2) (similar to) | upregulated anther stage 12 vs. anther stage 11, upregulated anther stage 12 vs. pollen |  |
| gene04349 | 35007427 | 35009609 | HOM03D001561 | ORTHO03D011485 | Phosphatidylinositol-3,4,5-trisphosphate 3-phosphatase and dual-specificity protein phosphatase PTEN (similar to) | upregulated pollen vs. anther stage 12 |  |
| gene04350 | 35010519 | 35011732 | HOM03D003094 | ORTHO03D247346 | Vesicle transport protein USE1 (probable) |  |  |
| gene04351 | 35015404 | 35016351 | HOM03D002103 | ORTHO03D043217 | Minor core protein (pV) (probable) |  |  |
| gene04352 | 35018895 | 35020322 | HOM03D000013 | ORTHO03D036685 | Putative ribonuclease H protein At1g65750 (probable) | upregulated anther stage 10 vs. anther stage 9, upregulated anther stage 11 vs. anther stage 10, upregulated anther stage 11 vs. anther stage 12 |  |
| gene04353 | 35021343 | 35026772 | HOM03D004691 | ORTHO03D003230 | Probable ubiquitin conjugation factor E4 (UB fusion protein 2-like) (putative) |  |  |
| gene04354 | 35027459 | 35030560 | HOM03D005079 | ORTHO03D004111 | tRNA guanosine-2'-O-methyltransferase (probable) |  |  |
| gene04355 | 35030996 | 35032121 | HOM03D000099 | ORTHO03D031519 | Anthocyanidin 5,3-O-glucosyltransferase (probable) | upregulated anther stage 6-7 vs. flower, upregulated anther stage 11 vs. anther stage 10, upregulated microspore vs. anther stage 10 |  |
| gene04356 | 35032945 | 35033610 | HOM03D005079 | ORTHO03D205682 | NADH-ubiquinone oxidoreductase chain 5 (probable) | upregulated anther stage 11 vs. anther stage 10 |  |
| gene04357 | 35036160 | 35037197 | HOM03D000099 | ORTHO03D031519 | Anthocyanidin 5,3-O-glucosyltransferase (probable) | upregulated microspore vs. anther stage 10 |  |
| gene04358 | 35038296 | 35040050 | HOM03D000096 | ORTHO03D185067 | Rho/Cdc42/Rac GTPase-activating protein RICS (probable) |  |  |
| gene04359 | 35045624 | 35047562 | HOM03D000099 | ORTHO03D008446 | Anthocyanidin 5,3-O-glucosyltransferase (probable) | upregulated microspore vs. anther stage 10 |  |
| gene04360 | 35047776 | 35052778 | HOM03D004214 | ORTHO03D003614 | Probable mitochondrial saccharopine dehydrogenase At5g39410 (SDH) (similar to) | upregulated anther stage 12 vs. pollen |  |
| gene04361 | 35053131 | 35059945 | HOM03D000633 | ORTHO03D002052 | Zinc finger MYM-type protein 1 (probable) | upregulated anther stage 12 vs. anther stage 11 |  |
| gene04362 | 35062241 | 35063924 | HOM03D007426 | ORTHO03D014729 | Blue-sensitive opsin (probable) |  |  |
| gene04363 | 35065539 | 35066133 | HOM03D007426 | ORTHO03D014729 | Integrase catalytic domain-containing protein KIAA1305 (probable) |  |  |
| gene04364 | 35070270 | 35072597 | HOM03D004214 | ORTHO03D003614 | Probable mitochondrial saccharopine dehydrogenase At5g39410 (SDH) (similar to) | upregulated anther stage 12 vs. pollen |  |
| gene04365 | 35076332 | 35078202 | HOM03D007426 | ORTHO03D014729 | ATP-dependent helicase/nuclease subunit A (probable) |  |  |
| gene04366 | 35084320 | 35090428 | HOM03D007426 | ORTHO03D014729 | Mitosis inhibitor protein kinase SWE1 (probable) |  |  |
| gene04367 | 35095149 | 35097444 | HOM03D000157 | ORTHO03D019081 | Putative AC9 transposase (probable) | upregulated microspore vs. anther stage 10 |  |
| gene04368 | 35099758 | 35100225 | HOM03D007426 | ORTHO03D037469 | Leucine-rich repeat-containing protein 3, Precursor (probable) |  |  |
| gene04369 | 35100483 | 35101215 | HOM03D024238 | ORTHO03D313939 | Probable mitochondrial tRNA-specific 2-thiouridylase 1 |  |  |
| gene04370 | 35101844 | 35105641 | HOM03D007426 | ORTHO03D014729 | Conserved oligomeric Golgi complex subunit 2 (COG complex subunit 2) (probable) |  |  |
| gene34750 | 35106745 | 35107359 | HOM03D000210 | ORTHO03D037155 | Adenosine deaminase (probable) |  |  |
| gene04371 | 35111897 | 35112286 | HOM03D001587 | ORTHO03D318014 | 2,3-bisphosphoglycerate-dependent phosphoglycerate mutase (probable) |  |  |
| gene04372 | 35112750 | 35115143 | HOM03D004214 | ORTHO03D003614 | Probable mitochondrial saccharopine dehydrogenase At5g39410 (SDH) (similar to) |  |  |
| gene04373 | 35115873 | 35118040 | HOM03D007426 | ORTHO03D014729 | Alanyl-tRNA synthetase (AlaRS) (probable) |  |  |
| gene04374 | 35118720 | 35119414 | HOM03D042743 | ORTHO03D192163 |  |  |  |
| gene04375 | 35122126 | 35122405 | HOM03D024238 | ORTHO03D244748 | Pre-mRNA-splicing factor cwf19 (probable) |  |  |
| gene04376 | 35122990 | 35126972 | HOM03D007426 | ORTHO03D014729 | Apolipoprotein A-IV (Apo-AIV), Precursor (probable) |  |  |
| gene04377 | 35128037 | 35129155 | HOM03D007426 | ORTHO03D014729 | Solute carrier family 12 member 5 (rKCC2) (probable) |  |  |
| gene04378 | 35131142 | 35132654 | HOM03D007426 | ORTHO03D014729 | Transmembrane and TPR repeat-containing protein CG5038 (probable) |  |  |
| gene04379 | 35133757 | 35134266 | HOM03D013520 | ORTHO03D030193 | Zinc finger protein jing homolog (probable) |  |  |
| gene04380 | 35134923 | 35136815 | HOM03D007426 | ORTHO03D014729 | Flap structure-specific endonuclease (probable) |  |  |
| gene04381 | 35137315 | 35137866 | HOM03D007426 | ORTHO03D014729 | Histidinol-phosphate aminotransferase 2 (probable) |  |  |
| gene04382 | 35140143 | 35142994 | HOM03D001587 | ORTHO03D051582 | Pumilio domain-containing protein P35G2.14 (probable) | upregulated anther stage 12 vs. anther stage 11 |  |
| gene04383 | 35146775 | 35147896 | HOM03D002350 | ORTHO03D008340 | UPF0301 protein Plut_0637 (probable) |  |  |
| gene04384 | 35148428 | 35150239 | HOM03D000001 | ORTHO03D069682 | Pentatricopeptide repeat-containing protein At3g29230 (putative) | upregulated pollen vs. anther stage 12 | 1 |
| gene04385 | 35150306 | 35154181 | HOM03D001465 | ORTHO03D003961 | Chorismate mutase, chloroplastic, Precursor (similar to) | upregulated anther stage 9 vs. anther stage 7-8, upregulated anther stage 12 vs. pollen, upregulated anther stage 10 vs. microspore |  |
| gene04386 | 35156526 | 35157696 | HOM03D000047 | ORTHO03D009975 | Lamin-like protein, Precursor (similar to) | upregulated anther stage 7-8 vs. anther stage 9, upregulated anther stage 12 vs. pollen, upregulated anther stage 10 vs. microspore |  |
| gene04387 | 35159354 | 35160657 | HOM03D000047 | ORTHO03D013487 | Lamin-like protein, Precursor (similar to) |  |  |
| gene04388 | 35161217 | 35162639 | HOM03D004266 | ORTHO03D079313 | Cell division topological specificity factor (probable) |  |  |
| gene04389 | 35162967 | 35163266 | HOM03D000387 | ORTHO03D272577 | Serine/threonine protein phosphatase 7 (probable) |  |  |
| gene04390 | 35163488 | 35166412 | HOM03D006596 | ORTHO03D007950 | E3 ubiquitin-protein ligase RNF181 (similar to) | upregulated anther stage 10 vs. microspore |  |
| gene04391 | 35168924 | 35171898 | HOM03D000029 | ORTHO03D000771 | Probable WRKY transcription factor 3 (putative),PlantTFDB=WRKY | upregulated anther stage 12 vs. pollen, upregulated microspore vs. anther stage 10 |  |
| gene04392 | 35173581 | 35182186 | HOM03D000092 | ORTHO03D000012 | Probable serine/threonine-protein kinase At1g54610 (similar to) | upregulated pollen vs. anther stage 12 |  |
| gene04393 | 35182529 | 35184769 | HOM03D001216 | ORTHO03D021507 | Threonyl-tRNA synthetase, mitochondrial (ThrRS), Precursor (putative) | upregulated anther stage 9 vs. anther stage 7-8, upregulated anther stage 9 vs. anther stage 10, upregulated anther stage 10 vs. anther stage 11, upregulated pollen vs. anther stage 12 | 3 |
| gene04394 | 35185316 | 35188365 | HOM03D000917 | ORTHO03D013681 |  | upregulated pollen vs. anther stage 12 |  |
| gene04395 | 35191599 | 35192454 | HOM03D023791 | ORTHO03D067260 | Solute carrier family 23 member 3 (similar to) |  |  |
| gene04396 | 35192517 | 35196636 | HOM03D001987 | ORTHO03D004590 | UPF0414 transmembrane protein C20orf30 (probable) |  |  |
| gene04397 | 35198842 | 35202243 | HOM03D000043 | ORTHO03D001541 | SNF1-related protein kinase catalytic subunit alpha KIN10 (AKIN10) (putative) | upregulated pollen vs. anther stage 12, upregulated anther stage 10 vs. microspore |  |
| gene04398 | 35203177 | 35205243 | HOM03D001397 | ORTHO03D001512 | Endo-1,3(4)-beta-glucanase 1 (Endo-1,4-beta-glucanase 1), Precursor (probable) | upregulated anther stage 11 vs. anther stage 10, upregulated anther stage 12 vs. pollen |  |
| gene04399 | 35206959 | 35209040 | HOM03D001397 | ORTHO03D001655 | Putative endo-1,3(4)-beta-glucanase 2 (Endo-1,4-beta-glucanase 2) (probable) | upregulated anther stage 12 vs. anther stage 11, upregulated anther stage 12 vs. pollen |  |
| gene04400 | 35209514 | 35210770 | HOM03D000011 | ORTHO03D011092 | Ethylene-responsive transcription factor RAP2-11 (similar to),PlantTFDB=ERF |  |  |
| gene04401 | 35213612 | 35218691 | HOM03D000297 | ORTHO03D000322 | fatm_Uncharacterized membrane protein | sporophyte specific, upregulated anther stage 12 vs. pollen, upregulated anther stage 10 vs. microspore |  |
| gene04402 | 35225119 | 35227393 | HOM03D001313 | ORTHO03D008691 | Vesicle transport v-SNARE 13 (AtVTI13) (putative) | upregulated pollen vs. anther stage 12 |  |
| gene04403 | 35227786 | 35229690 | HOM03D000001 | ORTHO03D110668 | Pentatricopeptide repeat-containing protein At5g15340, mitochondrial, Precursor (putative) |  | 1 |
| gene04404 | 35230643 | 35231491 | HOM03D000073 | ORTHO03D004122 | Expansin-A25 (AtEXPA25), Precursor (similar to) |  |  |
| gene04405 | 35232467 | 35235248 | HOM03D000018 | ORTHO03D277015 | F-box/kelch-repeat protein At3g06240 (probable) |  |  |
| gene04406 | 35236548 | 35242104 | HOM03D000002 | ORTHO03D014515 | Pentatricopeptide repeat-containing protein At5g15280 (probable) | upregulated anther stage 12 vs. anther stage 11, upregulated pollen vs. anther stage 12 | 1,2 |
| gene04407 | 35242345 | 35243742 | HOM03D008008 | ORTHO03D020965 | F-box/LRR-repeat protein 13 (similar to) |  |  |
| gene04408 | 35246538 | 35249389 | HOM03D000984 | ORTHO03D206113 | Sucrose-phosphate synthase 2 (similar to) |  |  |
| gene04409 | 35251518 | 35255301 | HOM03D000010 | ORTHO03D000448 | Putative disease resistance protein RGA3 (probable) |  | 4 |
| gene04410 | 35256217 | 35259299 | HOM03D000033 | ORTHO03D221233 | Probable ATP-dependent RNA helicase DDX47 |  |  |
| gene04411 | 35260000 | 35262877 | HOM03D003438 | ORTHO03D008120 | Glucosamine--fructose-6-phosphate aminotransferase [isomerizing] (probable) | upregulated anther stage 10 vs. anther stage 9, upregulated anther stage 11 vs. anther stage 12, upregulated microspore vs. anther stage 10 |  |
| gene04412 | 35263681 | 35266381 | HOM03D000030 | ORTHO03D004045 | Pectinesterase 31 (PE 31) (putative) | upregulated anther stage 10 vs. microspore |  |
| gene04413 | 35267239 | 35267604 | HOM03D003018 | ORTHO03D342396 |  |  |  |
| gene04414 | 35269014 | 35270948 | HOM03D003018 | ORTHO03D007097 |  | upregulated anther stage 12 vs. pollen |  |
| gene04415 | 35272992 | 35274410 | HOM03D000001 | ORTHO03D014321 | Pentatricopeptide repeat-containing protein At1g74630 (probable) | upregulated anther stage 11 vs. anther stage 12, upregulated microspore vs. anther stage 10 | 1 |
| gene04416 | 35275213 | 35276972 | HOM03D000623 | ORTHO03D012318 | Transmembrane emp24 domain-containing protein 10 (S31I125), Precursor (probable) | upregulated anther stage 12 vs. anther stage 11 |  |
| gene04417 | 35278332 | 35281848 | HOM03D000448 | ORTHO03D000155 | Putative phosphate transporter 1 (probable) | upregulated anther stage 12 vs. pollen |  |
| gene04418 | 35282806 | 35283568 | HOM03D000562 | ORTHO03D229294 | Putative F-box/LRR-repeat protein 23 (probable) |  |  |
| gene04419 | 35284715 | 35287389 | HOM03D000858 | ORTHO03D008910 | Xenotropic and polytropic retrovirus receptor 1 homolog (probable) | upregulated anther stage 12 vs. anther stage 11, upregulated pollen vs. anther stage 12 |  |
| gene04420 | 35288009 | 35291195 | HOM03D001317 | ORTHO03D001866 | Cystathionine gamma-synthase, chloroplastic (CGS), Precursor (putative) | sporophyte specific, upregulated anther stage 12 vs. pollen, upregulated anther stage 10 vs. microspore |  |
| gene04421 | 35295406 | 35298479 | HOM03D104619 | ORTHO03D319544 | Mastermind-like protein 3 (Mam-3) (probable) |  |  |
| gene04422 | 35299240 | 35302621 | HOM03D007907 | ORTHO03D032690 | Structural protein ORF567 (probable) |  |  |
| gene04423 | 35304198 | 35307054 | HOM03D000008 | ORTHO03D003285 | Transcription factor MYB39 (AtMYB39) (similar to),PlantTFDB=MYB | upregulated anther stage 11 vs. anther stage 12, sporophyte specific, upregulated anther stage 12 vs. pollen, upregulated anther stage 10 vs. microspore |  |
| gene04424 | 35316496 | 35317716 | HOM03D000015 | ORTHO03D013395 | Protein CUP-SHAPED COTYLEDON 2 (ANAC098) (similar to),PlantTFDB=NAC | upregulated anther stage 12 vs. pollen | 4 |
| gene04425 | 35324867 | 35326489 | HOM03D000001 | ORTHO03D076169 | Pentatricopeptide repeat-containing protein At5g15300 (similar to) |  | 1 |
| gene04426 | 35326906 | 35327930 | HOM03D000244 | ORTHO03D010396 | UPF0497 membrane protein 3 (similar to) |  |  |
| gene04427 | 35329131 | 35333334 | HOM03D000002 | ORTHO03D014515 | Pentatricopeptide repeat-containing protein At5g15280 (probable) |  | 1,2 |
| gene04428 | 35333588 | 35335412 | HOM03D008008 | ORTHO03D051783 | F-box/FBD/LRR-repeat protein At1g13570 (probable) |  |  |
| gene04429 | 35336355 | 35343303 | HOM03D000984 | ORTHO03D000250 | Sucrose-phosphate synthase 2 (similar to) | upregulated anther stage 12 vs. pollen |  |
| gene04430 | 35345221 | 35349806 | HOM03D000010 | ORTHO03D000448 | Putative disease resistance protein RGA3 (probable) |  | 4 |
| gene04431 | 35350146 | 35353908 | HOM03D008135 | ORTHO03D014313 | Reticulocyte-binding protein 3, Precursor (probable) | upregulated anther stage 9 vs. anther stage 7-8, upregulated anther stage 9 vs. anther stage 10 |  |
| gene04432 | 35360933 | 35363968 | HOM03D000393 | ORTHO03D069039 | KH domain-containing protein At4g18375 (probable) | upregulated anther stage 10 vs. microspore |  |
| gene04433 | 35365648 | 35368734 | HOM03D001156 | ORTHO03D000660 | Polypyrimidine tract-binding protein homolog 1 (similar to) |  |  |
| gene04434 | 35368879 | 35369564 | HOM03D001156 | ORTHO03D305489 | Polypyrimidine tract-binding protein homolog 1 (similar to) |  |  |
| gene04435 | 35373804 | 35375127 | HOM03D000073 | ORTHO03D008234 | Expansin-A1 (AtEXPA1), Precursor (similar to) |  |  |
| gene04436 | 35378052 | 35380778 | HOM03D000020 | ORTHO03D000047 | Probable disease resistance protein At5g63020 |  |  |
| gene04437 | 35384777 | 35386891 | HOM03D000001 | ORTHO03D101589 | Pentatricopeptide repeat-containing protein At5g39680 (putative) |  | 1 |
| gene04438 | 35387641 | 35388240 | HOM03D000053 | ORTHO03D007467 | Probable calcium-binding protein CML45 |  |  |
| gene04439 | 35389000 | 35391833 | HOM03D000052 | ORTHO03D008572 | Exopolygalacturonase, Precursor (probable) | upregulated anther stage 11 vs. anther stage 10 |  |
| gene04440 | 35393407 | 35393840 | HOM03D007736 | ORTHO03D013805 | Protein VP5 (probable) | upregulated anther stage 10 vs. anther stage 9, upregulated anther stage 11 vs. anther stage 10 |  |
| gene04441 | 35398134 | 35400788 | HOM03D000095 | ORTHO03D006621 | Dof zinc finger protein DOF5.2 (AtDOF5.2) (probable),PlantTFDB=Dof | upregulated pollen vs. anther stage 12 |  |
| gene04442 | 35404116 | 35404757 | HOM03D001800 | ORTHO03D003675 |  | upregulated anther stage 11 vs. anther stage 10, upregulated pollen vs. anther stage 12, upregulated microspore vs. anther stage 10 |  |
| gene04443 | 35407843 | 35409966 | HOM03D000001 | ORTHO03D033389 | Pentatricopeptide repeat-containing protein At5g50390, chloroplastic, Precursor (putative) | upregulated anther stage 12 vs. pollen | 1,3 |
| gene04444 | 35413462 | 35417771 | HOM03D000010 | ORTHO03D000448 | Putative disease resistance protein RGA3 (probable) |  | 4 |
| gene04445 | 35419758 | 35426811 | HOM03D008008 | ORTHO03D051783 | E3 ubiquitin-protein ligase RNF181 (probable) |  |  |
| gene04446 | 35429725 | 35429977 | HOM03D079292 | ORTHO03D323820 | Ena/VASP-like protein (probable) |  |  |
| gene04447 | 35430092 | 35430454 | HOM03D000067 | ORTHO03D028510 | RING-H2 finger protein ATL5P (probable) | upregulated anther stage 9 vs. anther stage 7-8, upregulated anther stage 10 vs. anther stage 11 |  |
| gene04448 | 35431061 | 35431652 | HOM03D030260 | ORTHO03D183501 | DNA-binding protein cre-1 (probable) |  |  |
| gene04449 | 35431946 | 35439581 | HOM03D005693 | ORTHO03D006293 | T-complex protein 1 subunit delta (TCP-1-delta) (probable) | upregulated pollen vs. anther stage 12 |  |
| gene04450 | 35441406 | 35443013 | HOM03D000002 | ORTHO03D008578 | Pentatricopeptide repeat-containing protein At5g15010, mitochondrial, Precursor (similar to) | upregulated microspore vs. anther stage 10 | 1,2 |
| gene04451 | 35444023 | 35444541 | HOM03D001465 | ORTHO03D161746 | Chorismate mutase, chloroplastic, Precursor (similar to) |  |  |
| gene04452 | 35446898 | 35456860 | HOM03D000412 | ORTHO03D017034 | Granule-bound starch synthase 2, chloroplastic/amyloplastic (GBSS-II), Precursor (similar to) | sporophyte specific, upregulated anther stage 12 vs. pollen, upregulated anther stage 10 vs. microspore |  |
| gene04453 | 35458031 | 35465543 | HOM03D000708 | ORTHO03D182270 | Cyclin-T1-4 (CycT1) (probable) |  |  |
| gene04454 | 35468075 | 35469447 | HOM03D008008 | ORTHO03D234425 | F-box/FBD/LRR-repeat protein At3g52680 (probable) |  |  |
| gene04455 | 35470564 | 35471292 | HOM03D000447 | ORTHO03D004668 | Protein bassoon (probable) | upregulated anther stage 11 vs. anther stage 10, upregulated anther stage 12 vs. anther stage 11, upregulated pollen vs. anther stage 12 |  |
| gene04456 | 35474106 | 35476559 | HOM03D000022 | ORTHO03D000967 | Cell division protease ftsH homolog 6, chloroplastic (AtFtsH6), Precursor (similar to) |  |  |
| gene04457 | 35476798 | 35477293 | HOM03D052160 | ORTHO03D186496 | Prostaglandin-H2 D-isomerase (PGD2 synthase), Precursor (probable) |  |  |
| gene04458 | 35480660 | 35481598 | HOM03D000542 | ORTHO03D019968 | E3 ubiquitin-protein ligase MARCH1 (MARCH-I) (probable) |  |  |
| gene04459 | 35483411 | 35484928 | HOM03D000001 | ORTHO03D011836 | Pentatricopeptide repeat-containing protein At2g36730 (putative) | upregulated anther stage 6-7 vs. flower | 1 |
| gene04460 | 35485939 | 35487683 | HOM03D000338 | ORTHO03D001373 | Vacuolar amino acid transporter 1 (probable) | upregulated anther stage 11 vs. anther stage 10, upregulated anther stage 11 vs. anther stage 12 |  |
| gene04461 | 35494141 | 35495485 | HOM03D000373 | ORTHO03D006274 | Aquaporin NIP2-1 (similar to) |  |  |
| gene04462 | 35496046 | 35499185 | HOM03D000091 | ORTHO03D339978 | RNA-binding protein with serine-rich domain 1-A (probable) | upregulated anther stage 6-7 vs. flower, upregulated anther stage 10 vs. anther stage 11, upregulated anther stage 11 vs. anther stage 12 |  |
| gene04463 | 35499331 | 35500826 | HOM03D000373 | ORTHO03D006274 | Aquaporin NIP2-1 (similar to) |  |  |
| gene03788 | 35515591 | 35521541 | HOM03D000338 | ORTHO03D167942 | Vacuolar amino acid transporter 4 (probable) |  |  |
| gene03789 | 35523218 | 35524505 | HOM03D000373 | ORTHO03D231500 | Aquaporin NIP2-1 (similar to) |  |  |
| gene03790 | 35525569 | 35529721 | HOM03D005223 | ORTHO03D037467 | Protein MCM10 homolog (probable) |  |  |
| gene03791 | 35532767 | 35533364 | HOM03D009256 | ORTHO03D054935 | Cullin-2 (CUL-2) (similar to) |  |  |
| gene03812 | 35543948 | 35552770 | HOM03D000373 | ORTHO03D006274 | Aquaporin NIP2-1 (similar to) |  |  |
| gene03813 | 35556499 | 35557661 | HOM03D000373 | ORTHO03D006274 | Aquaporin NIP2-1 (similar to) |  |  |
| gene03814 | 35572046 | 35578868 | HOM03D000209 | ORTHO03D226417 | Gibberellin-regulated protein 4, Precursor (probable) | upregulated anther stage 11 vs. anther stage 10, sporophyte specific, upregulated anther stage 12 vs. pollen, upregulated anther stage 10 vs. microspore |  |
| gene03815 | 35586199 | 35587281 | HOM03D000201 | ORTHO03D012503 | ZF-HD homeobox protein At5g65410 (probable),PlantTFDB=ZF-HD | upregulated anther stage 12 vs. anther stage 11, upregulated anther stage 12 vs. pollen |  |
| gene03816 | 35596577 | 35602252 | HOM03D000201 | ORTHO03D007695 | ZF-HD homeobox protein At4g24660 (AtHB-22) (probable),PlantTFDB=ZF-HD | upregulated anther stage 12 vs. anther stage 11, upregulated anther stage 12 vs. pollen |  |
| gene03817 | 35605493 | 35606965 | HOM03D000008 | ORTHO03D007466 | Myb-related protein 306 (probable),PlantTFDB=MYB |  |  |
| maker-LG6-augustus-gene-338.177 | 35607271 | 35607569 | NA | NA | uncharacterized protein |  | 5 |
| gene03818 | 35613864 | 35614661 | HOM03D013455 | ORTHO03D044509 | 60S ribosomal protein L12-2 (similar to) |  |  |
| gene03819 | 35617501 | 35620980 | HOM03D000886 | ORTHO03D004000 | Gelsolin-related protein of 125 kDa (GRP125) (probable) | upregulated anther stage 11 vs. anther stage 10 |  |
| gene03820 | 35621490 | 35622239 | HOM03D004009 | ORTHO03D051482 |  | upregulated anther stage 9 vs. anther stage 7-8, upregulated anther stage 10 vs. anther stage 11 |  |
| gene03821 | 35623586 | 35624414 | HOM03D080120 | ORTHO03D254802 | Carbamoyl-phosphate synthase large chain (probable) |  |  |
| gene03822 | 35624817 | 35625548 | HOM03D004009 | ORTHO03D051482 |  | upregulated anther stage 9 vs. anther stage 7-8 |  |
| gene03823 | 35626091 | 35628743 | HOM03D022319 | ORTHO03D067475 |  | upregulated anther stage 9 vs. anther stage 7-8 |  |
| gene03824 | 35630347 | 35631261 | HOM03D022319 | ORTHO03D067475 |  | upregulated anther stage 11 vs. anther stage 12 |  |
| gene03825 | 35632695 | 35635453 | HOM03D000580 | ORTHO03D019808 | Lysosomal Pro-X carboxypeptidase (PRCP), Precursor (probable) | upregulated anther stage 12 vs. anther stage 11 |  |
| gene03826 | 35635908 | 35636972 | HOM03D004009 | ORTHO03D211392 |  | upregulated anther stage 11 vs. anther stage 12 |  |
| gene03827 | 35637611 | 35638669 | HOM03D004009 | ORTHO03D229512 |  | upregulated anther stage 12 vs. pollen |  |
| gene03828 | 35639701 | 35640936 | HOM03D001448 | ORTHO03D239051 | 60S ribosomal protein L34 (putative) | upregulated anther stage 12 vs. pollen, upregulated anther stage 10 vs. microspore |  |
| gene03829 | 35641685 | 35645410 | HOM03D001303 | ORTHO03D010038 | Protease Do-like 8, chloroplastic, Precursor (putative) |  |  |
| gene03830 | 35645859 | 35646656 | HOM03D000067 | ORTHO03D057363 | E3 ubiquitin-protein ligase Praja1 (Praja-1) (similar to) | upregulated anther stage 9 vs. anther stage 7-8, upregulated anther stage 9 vs. anther stage 10 |  |
| gene03831 | 35647701 | 35648555 | HOM03D000067 | ORTHO03D057363 | E3 ubiquitin-protein ligase RNF181 (probable) | upregulated anther stage 10 vs. anther stage 11 |  |
| gene03832 | 35648713 | 35649491 | HOM03D062716 | ORTHO03D183547 | Uncharacterized protein HHRF5 (similar to) |  |  |
| gene03833 | 35655298 | 35656627 | HOM03D000015 | ORTHO03D010662 | Putative NAC domain-containing protein 9 (ANAC009) (similar to),PlantTFDB=NAC |  |  |
| gene03834 | 35658559 | 35659473 | HOM03D000290 | ORTHO03D299078 | Anthocyanin 5-aromatic acyltransferase (5AT) (probable) |  |  |
| gene03835 | 35660230 | 35661615 | HOM03D000290 | ORTHO03D001756 | Anthocyanin 5-aromatic acyltransferase (5AT) (probable) | upregulated anther stage 12 vs. pollen |  |
| gene03836 | 35663139 | 35663871 | HOM03D000233 | ORTHO03D204615 |  | upregulated anther stage 12 vs. anther stage 11 | 3 |
| gene03837 | 35667864 | 35668313 | HOM03D104613 | ORTHO03D313256 | Tryptophan synthase beta chain (probable) |  |  |
| gene03838 | 35668566 | 35669672 | HOM03D003769 | ORTHO03D031535 | Sentrin-specific protease 8 (probable) | upregulated anther stage 9 vs. anther stage 7-8, upregulated anther stage 9 vs. anther stage 10 |  |
| gene03839 | 35670477 | 35677037 | HOM03D006171 | ORTHO03D010975 | Heterogeneous nuclear ribonucleoprotein 27C (Hrb27-C) (probable) | upregulated anther stage 12 vs. pollen |  |
| gene03840 | 35678169 | 35678566 | HOM03D008951 | ORTHO03D020140 | Kinesin-like protein KIF20B (MPP1) (probable) |  |  |
| gene34751 | 35685710 | 35686015 | HOM03D011866 | ORTHO03D058709 | Endonuclease (probable) |  |  |
| gene03841 | 35689000 | 35691888 | HOM03D000984 | ORTHO03D162698 | Sucrose-phosphate synthase 2 (similar to) |  |  |
| gene03842 | 35694540 | 35698999 | HOM03D001791 | ORTHO03D051144 | Putative adenylate cyclase regulatory protein (probable) |  |  |
| gene03843 | 35699786 | 35701306 | HOM03D008951 | ORTHO03D020140 | 30S ribosomal protein S13 (probable) | upregulated anther stage 9 vs. anther stage 7-8 |  |
| gene03844 | 35701848 | 35704603 | HOM03D001791 | ORTHO03D022649 | Protein SET (probable) |  |  |
| gene03845 | 35704890 | 35712833 | HOM03D006614 | ORTHO03D009270 | Thyroid adenoma-associated protein homolog (probable) | upregulated anther stage 12 vs. pollen |  |
| gene03846 | 35716390 | 35718728 | HOM03D000002 | ORTHO03D220647 | Pentatricopeptide repeat-containing protein At5g15280 (probable) | upregulated microspore vs. anther stage 10 | 1,2 |
| gene03847 | 35719314 | 35723485 | HOM03D000010 | ORTHO03D000448 | Putative disease resistance protein RGA3 (probable) | upregulated pollen vs. anther stage 12 | 4 |
| gene03848 | 35725248 | 35731944 | HOM03D000287 | ORTHO03D000810 | Cullin-3 (CUL-3) (similar to) |  |  |
| gene03849 | 35733590 | 35735086 | HOM03D001432 | ORTHO03D000609 | 40S ribosomal protein S9-2 | upregulated anther stage 7-8 vs. anther stage 9, upregulated anther stage 12 vs. pollen |  |
| gene03850 | 35735235 | 35736453 | HOM03D007969 | ORTHO03D015571 | Homeobox protein bagpipe (probable) | upregulated anther stage 12 vs. anther stage 11 |  |
| gene03851 | 35737394 | 35739410 | HOM03D000159 | ORTHO03D089175 | Tubulin alpha chain | upregulated anther stage 11 vs. anther stage 12, upregulated anther stage 10 vs. microspore |  |
| gene03852 | 35741838 | 35742340 | HOM03D000005 | ORTHO03D262493 |  |  |  |
| gene03853 | 35751596 | 35757996 | HOM03D000078 | ORTHO03D088668 | ABC transporter B family member 19 (ABC transporter ABCB.19) (putative) |  |  |
| gene01244 | 35771672 | 35777513 | HOM03D006171 | ORTHO03D010975 | Ankyrin repeat domain-containing protein 1 (probable) | upregulated pollen vs. anther stage 12 |  |
| gene01245 | 35778271 | 35779818 | HOM03D008951 | ORTHO03D020140 | DNA polymerase alpha catalytic subunit (probable) |  |  |
| gene01246 | 35783689 | 35784101 | HOM03D002118 | ORTHO03D335137 | Maturase K (probable) |  |  |
| gene01247 | 35786687 | 35787236 | HOM03D006171 | ORTHO03D030572 | Probable tRNA (uracil-O(2)-)-methyltransferase (similar to) | upregulated pollen vs. anther stage 12 |  |
| gene01248 | 35788114 | 35788638 | HOM03D007843 | ORTHO03D152522 | GTP-binding protein lepA (probable) | upregulated anther stage 6-7 vs. flower |  |
| gene01249 | 35790263 | 35791802 | HOM03D008951 | ORTHO03D020140 | Glycophorin-binding protein (probable) | upregulated pollen vs. anther stage 12 |  |
| gene01250 | 35795468 | 35796603 | HOM03D008951 | ORTHO03D020140 | Mental retardation GTPase activating protein homolog 1 (probable) |  |  |
| gene01251 | 35797372 | 35799845 | HOM03D004445 | ORTHO03D004571 | 3-dehydroquinate synthase (DHQ synthase) (probable) |  |  |
| gene01252 | 35800498 | 35802826 | HOM03D001010 | ORTHO03D002264 | Aldose 1-epimerase (probable) | upregulated anther stage 10 vs. microspore |  |
| gene01253 | 35804969 | 35806913 | HOM03D002113 | ORTHO03D029458 | Quinohaemoprotein ethanol dehydrogenase type-1, Precursor (probable) | upregulated anther stage 9 vs. anther stage 7-8 |  |
| gene01254 | 35808397 | 35810699 | HOM03D002113 | ORTHO03D029458 | Quinohaemoprotein ethanol dehydrogenase type-1, Precursor (probable) |  |  |
| gene01255 | 35811002 | 35824634 | HOM03D002113 | ORTHO03D081753 | GTP-binding protein GUF1 homolog (similar to) |  |  |
| gene33924 | 35816340 | 35816648 | HOM03D002113 | ORTHO03D285826 | Alcohol dehydrogenase [acceptor], Precursor (probable) | upregulated anther stage 9 vs. anther stage 7-8 |  |
| gene01256 | 35825535 | 35827465 | HOM03D000029 | ORTHO03D010187 | Probable WRKY transcription factor 72,PlantTFDB=WRKY | upregulated anther stage 10 vs. anther stage 9, upregulated anther stage 10 vs. anther stage 11 |  |
| gene01257 | 35844154 | 35847675 | HOM03D000675 | ORTHO03D008353 | 2-aminoethanethiol dioxygenase (probable) | upregulated microspore vs. anther stage 10 |  |
| gene01258 | 35854354 | 35854561 | HOM03D044414 | ORTHO03D199108 |  |  |  |
| gene01259 | 35854695 | 35858009 | HOM03D003732 | ORTHO03D003201 | V-type proton ATPase subunit d2 (V-ATPase subunit d2) |  |  |
| gene01260 | 35858654 | 35861725 | HOM03D006466 | ORTHO03D009469 | F-box/WD repeat-containing protein sel-10 (probable) |  |  |
| gene01261 | 35862540 | 35864576 | HOM03D005644 | ORTHO03D012305 | 50S ribosomal protein L2 (probable) | upregulated anther stage 12 vs. pollen |  |
| gene01262 | 35865802 | 35867469 | HOM03D000142 | ORTHO03D005253 | Ethylene-responsive transcription factor WRI1 (similar to) |  |  |
| gene01263 | 35870030 | 35872351 | HOM03D002656 | ORTHO03D229292 | FACT complex subunit SSRP1 (similar to) | upregulated microspore vs. anther stage 10 |  |
| gene01264 | 35872445 | 35875529 | HOM03D002656 | ORTHO03D001936 | FACT complex subunit SSRP1 (similar to) | upregulated microspore vs. anther stage 10 |  |
| gene01265 | 35878355 | 35883478 | HOM03D002656 | ORTHO03D001936 | FACT complex subunit SSRP1 (similar to) | upregulated microspore vs. anther stage 10 |  |
| gene01266 | 35884139 | 35885844 | HOM03D000139 | ORTHO03D000235 | Probable pectate lyase P59, Precursor (putative) | upregulated pollen vs. anther stage 12 |  |
| gene01267 | 35887963 | 35889777 | HOM03D000385 | ORTHO03D007434 | Putative auxin efflux carrier component 5 (AtPIN5) |  |  |
| gene01268 | 35890772 | 35893504 | HOM03D000594 | ORTHO03D069487 | Mitochondrial outer membrane protein porin of 34 kDa (VDAC) (putative) | upregulated anther stage 12 vs. pollen |  |
| gene01269 | 35895204 | 35902691 | HOM03D003325 | ORTHO03D053337 | Protein kinase APK1A, chloroplastic, Precursor (similar to) | upregulated anther stage 12 vs. pollen |  |
| gene01270 | 35902887 | 35910130 | HOM03D006135 | ORTHO03D164439 | F-box protein At3g61340 (probable) | upregulated anther stage 9 vs. anther stage 10, upregulated anther stage 10 vs. anther stage 11 | 3 |
| gene01271 | 35910614 | 35912841 | HOM03D035486 | ORTHO03D193666 | 26S proteasome non-ATPase regulatory subunit 13 (probable) |  |  |
| gene01272 | 35912979 | 35913230 | HOM03D088049 | ORTHO03D237380 |  |  |  |
| gene01273 | 35914245 | 35915449 | HOM03D009773 | ORTHO03D032519 | GPI ethanolamine phosphate transferase 1 (probable) | upregulated microspore vs. anther stage 10 |  |
| gene33925 | 35918104 | 35918977 | HOM03D009773 | ORTHO03D032519 | Telomere length regulator protein rif1 (probable) |  |  |
| gene01274 | 35920798 | 35921319 | HOM03D007976 | ORTHO03D016673 | Kininogen-1 light chain, Precursor (similar to) | upregulated microspore vs. anther stage 10 |  |
| gene01275 | 35923495 | 35924016 | HOM03D007976 | ORTHO03D016673 | UPF0658 Golgi apparatus membrane protein C1952.10c (probable) |  |  |
| gene01276 | 35924081 | 35925321 | HOM03D017289 | ORTHO03D320461 |  |  |  |
| gene01277 | 35925366 | 35928905 | HOM03D006069 | ORTHO03D007651 | Protein midA, mitochondrial, Precursor (probable) |  |  |
| gene01278 | 35929382 | 35929992 | HOM03D000127 | ORTHO03D007815 | Thioredoxin H-type 2 (Trx-H-2) (similar to) |  |  |
| gene01279 | 35930411 | 35933860 | HOM03D000294 | ORTHO03D305953 | Plasma membrane ATPase 1 (similar to) |  |  |
| gene01280 | 35934451 | 35935047 | HOM03D000127 | ORTHO03D007815 | Thioredoxin H-type 2 (Trx-H-2) (similar to) | upregulated anther stage 12 vs. pollen |  |
| gene01281 | 35935843 | 35940697 | HOM03D000294 | ORTHO03D000024 | Plasma membrane ATPase 1 (putative) | upregulated anther stage 12 vs. anther stage 11, upregulated anther stage 12 vs. pollen |  |
| gene01282 | 35942251 | 35957933 | HOM03D002180 | ORTHO03D001301 | Inositol hexakisphosphate and diphosphoinositol-pentakisphosphate kinase 2 (mmVIP2) (probable) |  |  |
| gene01283 | 35958139 | 35959612 | HOM03D000001 | ORTHO03D012060 | Pentatricopeptide repeat-containing protein At3g28660 (similar to) | upregulated anther stage 6-7 vs. flower | 1 |
| gene01284 | 35960244 | 35961404 | HOM03D001022 | ORTHO03D012633 | Nitric oxide synthase (N-NOS) (probable) | upregulated pollen vs. anther stage 12 |  |
| gene01285 | 35962871 | 35965662 | HOM03D001022 | ORTHO03D309465 | Alpha-S1-casein, Precursor (probable) | upregulated pollen vs. anther stage 12 |  |
| gene01286 | 35967264 | 35968284 | HOM03D000083 | ORTHO03D018650 | LOB domain-containing protein 27 (AS2-like protein 29) (probable),PlantTFDB=LBD | upregulated anther stage 11 vs. anther stage 10, upregulated anther stage 12 vs. pollen |  |
| gene01287 | 35970838 | 35978251 | HOM03D000102 | ORTHO03D086475 | Pleiotropic drug resistance protein 3 (putative) |  |  |
| gene01288 | 35979986 | 35985952 | HOM03D000102 | ORTHO03D086476 | ABC transporter G family member 37 (ABC transporter ABCG.37) (similar to) | upregulated anther stage 12 vs. anther stage 11, upregulated pollen vs. anther stage 12 |  |
| gene01289 | 35987733 | 35988197 | HOM03D005476 | ORTHO03D005707 | Protein SYS1 homolog (probable) |  |  |
| gene01290 | 35989486 | 35993254 | HOM03D002313 | ORTHO03D000735 | HIPL1 protein, Precursor (similar to) |  |  |
| gene01291 | 35993333 | 35993710 | HOM03D002313 | ORTHO03D216938 | HIPL2 protein, Precursor (similar to) |  |  |
| gene01292 | 35993916 | 35998602 | HOM03D000005 | ORTHO03D103807 | TMV resistance protein N (probable) |  |  |
| gene01293 | 35998868 | 36000403 | HOM03D000005 | ORTHO03D335421 | TMV resistance protein N (similar to) | upregulated microspore vs. anther stage 10 | 4 |
| augustus_masked-LG6-processed-gene-17.24 | 36000730 | 36001643 | NA | NA | homolog of HIPL1 protein |  | 5 |
| gene01294 | 36002211 | 36003827 | HOM03D000005 | ORTHO03D103808 | TMV resistance protein N (probable) | upregulated anther stage 11 vs. anther stage 10, upregulated anther stage 12 vs. pollen |  |
| gene01295 | 36004309 | 36008892 | HOM03D000005 | ORTHO03D008946 | Pentatricopeptide repeat-containing protein At5g39980, chloroplastic, Precursor (similar to) |  | 1,4 |
| gene01296 | 36009621 | 36012577 | HOM03D000312 | ORTHO03D000604 | Xylosyltransferase 1 (probable) | upregulated anther stage 12 vs. pollen |  |
| gene01297 | 36017577 | 36027975 | HOM03D014931 | ORTHO03D295884 | Carbamoyl-phosphate synthase arginine-specific large chain (probable) |  |  |
| gene01298 | 36032723 | 36033772 | HOM03D003235 | ORTHO03D002846 | Chemotaxis response regulator protein-glutamate methylesterase (probable) |  |  |
| gene01299 | 36040046 | 36040369 | HOM03D007504 | ORTHO03D062047 | Protein DLN-1 (probable) |  |  |
| gene01300 | 36041561 | 36041789 | HOM03D079558 | ORTHO03D173971 |  |  |  |
| gene01301 | 36042377 | 36042760 | HOM03D036013 | ORTHO03D333340 | TATA element modulatory factor (TMF) (probable) |  |  |
| gene01302 | 36043791 | 36045209 | HOM03D000117 | ORTHO03D000131 | Probable mitochondrial chaperone BCS1-B | upregulated anther stage 7-8 vs. anther stage 9 |  |
| gene01303 | 36046457 | 36048046 | HOM03D000117 | ORTHO03D000131 | Probable mitochondrial chaperone BCS1-B |  |  |
| gene01304 | 36049285 | 36050727 | HOM03D000117 | ORTHO03D000131 | Probable mitochondrial chaperone bcs1 |  |  |
| gene01305 | 36056175 | 36056693 | HOM03D000018 | ORTHO03D064517 | Cytoplasmic dynein 2 heavy chain 1 (probable) |  |  |
| gene01306 | 36058679 | 36060280 | HOM03D000117 | ORTHO03D000131 | Probable mitochondrial chaperone bcs1 |  |  |
| gene01307 | 36062498 | 36063900 | HOM03D000018 | ORTHO03D064517 | F-box protein At4g12560 (probable) | upregulated microspore vs. anther stage 10 |  |
| gene01308 | 36065611 | 36067889 | HOM03D000106 | ORTHO03D009076 | Thaumatin-like protein, Precursor (probable) | upregulated anther stage 12 vs. anther stage 11 |  |
| gene01309 | 36067926 | 36069853 | HOM03D000076 | ORTHO03D000020 | Protein kinase PVPK-1 (similar to) |  |  |
| gene01310 | 36072803 | 36074320 | HOM03D000422 | ORTHO03D000558 | Glycerol-3-phosphate acyltransferase 6 (AtGPAT6) (putative) |  |  |
| gene01311 | 36074908 | 36084374 | HOM03D000319 | ORTHO03D057766 | Transcription factor MYB32 (AtMYB32) (similar to),PlantTFDB=MYB |  |  |
| gene01312 | 36087782 | 36091841 | HOM03D006135 | ORTHO03D348146 | Putative F-box protein At4g22170 (probable) |  |  |
| gene01313 | 36093555 | 36096339 | HOM03D000917 | ORTHO03D002692 | Mating-type locus allele B3 protein (probable) |  |  |
| gene01314 | 36097517 | 36099414 | HOM03D004775 | ORTHO03D005109 | Putative rRNA methyltransferase ylbH (probable) |  |  |
| gene01315 | 36103153 | 36104342 | HOM03D004052 | ORTHO03D013518 | Tripartite motif-containing protein 48 (probable) | upregulated anther stage 11 vs. anther stage 12 |  |
| gene01316 | 36107901 | 36109691 | HOM03D000006 | ORTHO03D000894 | Probable inactive receptor kinase At1g27190, Precursor (putative) | upregulated anther stage 12 vs. pollen |  |
| gene01317 | 36112721 | 36115819 | HOM03D002813 | ORTHO03D009661 | Tropomyosin-2 (TMII) (probable) |  |  |
| gene01318 | 36116964 | 36119289 | HOM03D000034 | ORTHO03D244542 | Aspartic proteinase nepenthesin-1, Precursor (probable) | upregulated pollen vs. anther stage 12 |  |
| gene01319 | 36119979 | 36120464 | HOM03D007611 | ORTHO03D012836 | Calcium-transporting ATPase 1, chloroplastic, Precursor (probable) |  |  |
| gene01320 | 36122001 | 36124432 | HOM03D000282 | ORTHO03D012010 | Type I inositol-1,4,5-trisphosphate 5-phosphatase CVP2 (similar to) | upregulated anther stage 11 vs. anther stage 10, upregulated anther stage 12 vs. pollen |  |
| gene01321 | 36124915 | 36127484 | HOM03D006292 | ORTHO03D008899 | Probable DNA double-strand break repair rad50 ATPase |  |  |
| gene01322 | 36130390 | 36134747 | HOM03D000078 | ORTHO03D088665 | ABC transporter B family member 15 (ABC transporter ABCB.15) (putative) | upregulated anther stage 10 vs. anther stage 9, upregulated anther stage 11 vs. anther stage 10, upregulated anther stage 12 vs. pollen, upregulated microspore vs. anther stage 10 |  |
| gene01323 | 36141043 | 36153925 | HOM03D000078 | ORTHO03D088664 | ABC transporter B family member 15 (ABC transporter ABCB.15) (similar to) | upregulated anther stage 10 vs. anther stage 9 |  |
| gene01324 | 36159140 | 36162167 | HOM03D000387 | ORTHO03D259535 | Paired amphipathic helix protein Sin3 (AtSin3) (similar to) | upregulated anther stage 10 vs. microspore |  |
| gene01325 | 36164026 | 36178549 | HOM03D000387 | ORTHO03D000294 | Paired amphipathic helix protein Sin3 (AtSin3) (probable) | upregulated pollen vs. anther stage 12 |  |
| gene01326 | 36179531 | 36181077 | HOM03D000033 | ORTHO03D000129 | Eukaryotic initiation factor 4A-3 (eIF-4A-3) (putative) |  |  |
| gene01327 | 36181643 | 36182860 | HOM03D000018 | ORTHO03D157911 | Putative F-box only protein 9 (probable) | upregulated anther stage 10 vs. anther stage 11 |  |
| gene01328 | 36183238 | 36185354 | HOM03D002819 | ORTHO03D008728 | Cell division cycle protein 48 homolog MJ1156 (probable) | sporophyte specific, upregulated anther stage 12 vs. pollen, upregulated anther stage 10 vs. microspore |  |
| gene01329 | 36186942 | 36189307 | HOM03D002499 | ORTHO03D000893 | Protein SEC13 homolog (similar to) | upregulated pollen vs. anther stage 12, upregulated anther stage 10 vs. microspore |  |
| gene20843 | 36202067 | 36204706 | HOM03D002591 | ORTHO03D317093 | RCC1 and BTB domain-containing protein 2 (probable) |  |  |
| gene20844 | 36210719 | 36211724 | HOM03D058542 | ORTHO03D272913 | Tubulin polyglutamylase TTLL6 (probable) |  |  |
| genemark-LG7-processed-gene-166.71 | 36213457 | 36214432 | NA | NA | uncharacterized protein |  | 5 |
| gene20845 | 36216113 | 36216664 | HOM03D000167 | ORTHO03D186108 | Disease resistance response protein 206 (probable) |  |  |
| gene20846 | 36226404 | 36236097 | HOM03D000052 | ORTHO03D296448 | Polygalacturonase (PG), Precursor (similar to) |  |  |
| gene34101 | 36227502 | 36228220 | HOM03D000263 | ORTHO03D317649 | F-box protein At3g56470 (probable) | upregulated anther stage 9 vs. anther stage 7-8, upregulated anther stage 9 vs. anther stage 10, upregulated pollen vs. anther stage 12 | 3 |
| gene20847 | 36238399 | 36243251 | HOM03D000117 | ORTHO03D002167 | Probable mitochondrial chaperone bcs1 | upregulated anther stage 10 vs. microspore |  |
| gene20848 | 36261058 | 36261719 | HOM03D045511 | ORTHO03D141969 | Defensin-like protein 156 (Protein LCR21), Precursor (probable) |  |  |
| gene20849 | 36263410 | 36277350 | HOM03D002885 | ORTHO03D297563 | 50S ribosomal protein L30P (probable) | upregulated anther stage 10 vs. microspore |  |
| gene28320 | 36295511 | 36304808 | HOM03D000640 | ORTHO03D012191 | Transcription factor bHLH135 (bHLH 135) (similar to),PlantTFDB=bHLH | upregulated anther stage 12 vs. anther stage 11, upregulated anther stage 12 vs. pollen |  |
| gene28321 | 36307776 | 36309203 | HOM03D000259 | ORTHO03D006804 | fatm_Glutaredoxin domain-containing cysteine-rich protein CG31559 | upregulated pollen vs. anther stage 12 |  |
| gene28322 | 36318644 | 36328468 | HOM03D000003 | ORTHO03D001356 | Protein kinase APK1A, chloroplastic, Precursor (similar to),PlantTFDB=HD-ZIP | upregulated anther stage 10 vs. anther stage 11 |  |
| gene28323 | 36330188 | 36331646 | HOM03D008951 | ORTHO03D020140 | Calcium-binding protein CML38 (probable) | upregulated pollen vs. anther stage 12, upregulated anther stage 10 vs. microspore |  |
| gene28324 | 36333371 | 36338424 | HOM03D014501 | ORTHO03D063796 | Pentatricopeptide repeat-containing protein At4g35850, mitochondrial, Precursor (similar to) |  | 1 |
| gene28325 | 36340985 | 36341332 | HOM03D007514 | ORTHO03D017028 |  |  |  |
| gene28326 | 36341474 | 36352594 | HOM03D006171 | ORTHO03D010975 | Tankyrase-2 (TANK2) (probable) |  |  |
| gene28327 | 36355602 | 36356012 | HOM03D008951 | ORTHO03D058559 | Cation channel sperm-associated protein 1 (CatSper1) (probable) | upregulated pollen vs. anther stage 12 |  |
| gene28328 | 36357616 | 36358026 | HOM03D008951 | ORTHO03D058559 | Cation channel sperm-associated protein 1 (CatSper1) (probable) | upregulated pollen vs. anther stage 12 |  |
| gene28329 | 36358834 | 36361130 | HOM03D000033 | ORTHO03D000129 | DEAD-box ATP-dependent RNA helicase 2 (similar to) | upregulated anther stage 9 vs. anther stage 7-8, upregulated anther stage 10 vs. anther stage 11, upregulated pollen vs. anther stage 12 |  |
| gene28330 | 36363271 | 36367369 | HOM03D000644 | ORTHO03D004403 | Alpha-mannosidase 2 (AMAN II) (probable) |  |  |
| gene28331 | 36368625 | 36374103 | HOM03D002679 | ORTHO03D000993 | Mannose-1-phosphate guanyltransferase alpha (probable) | upregulated anther stage 12 vs. pollen |  |
| gene28332 | 36374628 | 36377916 | HOM03D000025 | ORTHO03D000043 | Probable disease resistance RPP8-like protein 2 |  |  |
| gene28333 | 36378791 | 36381379 | HOM03D006776 | ORTHO03D025831 | Zinc transporter 5 (ZnT-5) (probable) | upregulated anther stage 12 vs. pollen, upregulated microspore vs. anther stage 10 |  |
| gene28334 | 36385984 | 36388059 | HOM03D000026 | ORTHO03D002171 | Peptide transporter PTR5 (probable) |  |  |
| gene28335 | 36388668 | 36391998 | HOM03D000430 | ORTHO03D009854 | Metalloendoproteinase 1, Precursor (probable) |  |  |
| gene28336 | 36392808 | 36393161 | HOM03D014226 | ORTHO03D032952 | Polycomb group protein Pc (Protein polycomb) (probable) |  |  |
| gene28337 | 36393208 | 36394893 | HOM03D000430 | ORTHO03D306364 | Metalloendoproteinase 1, Precursor (probable) | upregulated anther stage 12 vs. anther stage 11, upregulated anther stage 12 vs. pollen |  |
| gene28338 | 36395950 | 36397099 | HOM03D000430 | ORTHO03D240002 | Metalloendoproteinase 1, Precursor (probable) |  |  |
| gene28339 | 36399196 | 36400353 | HOM03D000430 | ORTHO03D009854 | Metalloendoproteinase 1, Precursor (probable) |  |  |
| gene28340 | 36402299 | 36405121 | HOM03D007598 | ORTHO03D011201 | Probable serine/threonine-protein kinase irlA |  |  |
| gene28341 | 36405461 | 36408934 | HOM03D006946 | ORTHO03D007873 | Protein CREG1, Precursor (probable) | upregulated anther stage 12 vs. anther stage 11, upregulated anther stage 12 vs. pollen |  |
| gene28342 | 36410233 | 36415540 | HOM03D000398 | ORTHO03D033521 | Chloroplastic group IIA intron splicing facilitator CRS1, chloroplastic, Precursor (probable) |  |  |
| gene28343 | 36420531 | 36427308 | HOM03D005727 | ORTHO03D004845 | Ferredoxin-thioredoxin reductase catalytic chain, chloroplastic (FTR-C), Precursor (similar to) |  |  |
| gene28344 | 36427712 | 36428287 | HOM03D002247 | ORTHO03D006285 | Zinc finger AN1 domain-containing stress-associated protein 12 (AtSAP12) (putative) | upregulated anther stage 9 vs. anther stage 10, upregulated pollen vs. anther stage 12 |  |
| gene28345 | 36431060 | 36432730 | HOM03D000061 | ORTHO03D008847 | U-box domain-containing protein 40 (similar to) |  |  |
| gene28346 | 36433025 | 36436716 | HOM03D000371 | ORTHO03D000184 | Probable disease resistance protein At5g66900 |  |  |
| gene28347 | 36438371 | 36441893 | HOM03D000371 | ORTHO03D000184 | Probable disease resistance protein At5g66900 |  |  |
| gene28348 | 36443738 | 36455021 | HOM03D000371 | ORTHO03D000184 | Probable disease resistance protein At5g66900 | upregulated pollen vs. anther stage 12 |  |
| gene28349 | 36456465 | 36459829 | HOM03D000371 | ORTHO03D000184 | Probable disease resistance protein At5g66900 |  |  |
| gene28350 | 36463301 | 36464272 | HOM03D000014 | ORTHO03D007130 | Peroxidase 63 (Atperox P63), Precursor (putative) | upregulated anther stage 11 vs. anther stage 12, sporophyte specific, upregulated anther stage 12 vs. pollen, upregulated anther stage 10 vs. microspore |  |
| gene28351 | 36465815 | 36476461 | HOM03D001021 | ORTHO03D000651 | Protein teflon (probable) | sporophyte specific, upregulated anther stage 12 vs. pollen, upregulated anther stage 10 vs. microspore |  |
| augustus_masked-LG4-processed-gene-102.34 | 36478774 | 36480755 | NA | NA | uncharacterized protein |  | 5 |
| gene28352 | 36481596 | 36490608 | HOM03D001021 | ORTHO03D000651 | GPI ethanolamine phosphate transferase 1 (probable) | upregulated anther stage 12 vs. anther stage 11, upregulated anther stage 12 vs. pollen |  |
| gene28353 | 36491354 | 36492249 | HOM03D000209 | ORTHO03D046714 | Gibberellin-regulated protein 1, Precursor (probable) | upregulated anther stage 11 vs. anther stage 10 |  |
| gene28354 | 36493386 | 36495700 | HOM03D013254 | ORTHO03D347237 | Snakin-2, Precursor (probable) | upregulated anther stage 12 vs. anther stage 11, upregulated pollen vs. anther stage 12 |  |
| gene28355 | 36497685 | 36498681 | HOM03D040808 | ORTHO03D165032 | Outer membrane protein icsA translocator, Precursor (similar to) |  |  |
| gene28356 | 36500888 | 36503771 | HOM03D106534 | ORTHO03D147067 |  | upregulated anther stage 11 vs. anther stage 10 |  |
| gene28357 | 36506431 | 36508118 | HOM03D106236 | ORTHO03D256668 | DNA-directed RNA polymerase subunit beta' (RNAP subunit beta') (probable) |  |  |
| gene28358 | 36509879 | 36510862 | HOM03D000209 | ORTHO03D018390 | Snakin-2, Precursor (similar to) | upregulated anther stage 10 vs. anther stage 9, upregulated anther stage 11 vs. anther stage 12, upregulated anther stage 10 vs. microspore |  |
| gene28359 | 36512848 | 36520541 | HOM03D000309 | ORTHO03D000407 | Xyloglucan glycosyltransferase 4 (AtCslC4) (similar to) | upregulated anther stage 12 vs. pollen |  |
| gene28360 | 36527961 | 36529189 | HOM03D002238 | ORTHO03D336374 | BAH and coiled-coil domain-containing protein 1 (probable) | upregulated microspore vs. anther stage 10 |  |
| gene28361 | 36529277 | 36533608 | HOM03D002238 | ORTHO03D109695 | Dentin sialoprotein, Precursor (probable) | upregulated pollen vs. anther stage 12 |  |
| gene28362 | 36535816 | 36538926 | HOM03D000042 | ORTHO03D002868 | Histidinol-phosphate aminotransferase 1 (probable) | upregulated anther stage 12 vs. anther stage 11 |  |
| gene28363 | 36555219 | 36556738 | HOM03D000371 | ORTHO03D001018 | Probable disease resistance protein At5g66900 |  |  |
| gene28364 | 36556931 | 36558134 | HOM03D000371 | ORTHO03D056228 | Probable disease resistance protein At5g66910 |  |  |
| gene28365 | 36562073 | 36562387 | HOM03D000042 | ORTHO03D170760 | Hexon protein (probable) |  |  |
| gene28366 | 36563440 | 36565587 | HOM03D000371 | ORTHO03D309526 | Probable disease resistance protein At5g66900 |  |  |
| gene28367 | 36565777 | 36566895 | HOM03D000371 | ORTHO03D056228 | Probable disease resistance protein At5g66910 |  |  |
| gene28368 | 36571965 | 36572354 | HOM03D000371 | ORTHO03D202583 | Probable disease resistance protein At5g66910 |  |  |
| gene28369 | 36573321 | 36578045 | HOM03D000042 | ORTHO03D002868 | 30S ribosomal protein S12P (probable) | upregulated anther stage 12 vs. anther stage 11, upregulated pollen vs. anther stage 12 |  |
| gene28370 | 36579041 | 36581976 | HOM03D000371 | ORTHO03D000184 | Probable disease resistance protein At5g66900 |  |  |
| gene28371 | 36584388 | 36584939 | HOM03D004959 | ORTHO03D004194 | UPF0477 protein BcerKBAB4_1135 (probable) | upregulated anther stage 11 vs. anther stage 12, upregulated anther stage 12 vs. pollen |  |
| gene28372 | 36586848 | 36590258 | HOM03D000718 | ORTHO03D001187 | Protease Do-like 9 (similar to) | upregulated anther stage 12 vs. pollen |  |
| gene28373 | 36591308 | 36593811 | HOM03D000718 | ORTHO03D001187 | Protease Do-like 9 (similar to) |  |  |
| gene28374 | 36594658 | 36596758 | HOM03D000049 | ORTHO03D009826 | Auxin-induced protein 5NG4 (probable) | upregulated anther stage 12 vs. anther stage 11, upregulated anther stage 12 vs. pollen |  |
| gene28375 | 36598628 | 36603866 | HOM03D000049 | ORTHO03D011404 | Auxin-induced protein 5NG4 (probable) | upregulated anther stage 9 vs. anther stage 7-8 |  |
| gene28376 | 36603905 | 36605050 | HOM03D000152 | ORTHO03D032140 | Protein RUPTURED POLLEN GRAIN 1 (probable) | upregulated anther stage 9 vs. anther stage 7-8, upregulated anther stage 11 vs. anther stage 12, Protein RUPTURED POLLEN GRAIN 1 (probable) |  |
| gene28377 | 36606879 | 36607976 | HOM03D000152 | ORTHO03D032140 | Protein RUPTURED POLLEN GRAIN 1 (probable) | upregulated anther stage 9 vs. anther stage 7-8, upregulated anther stage 10 vs. anther stage 11, upregulated anther stage 11 vs. anther stage 12, upregulated anther stage 10 vs. microspore, Protein RUPTURED POLLEN GRAIN 1 (probable) |  |
