# Supplementary material for: Multilocus Sex Determination Revealed in Two Populations of Gynodioecious Wild Strawberry, Fragaria vesca subsp. bracteata
Source: G3 (Bethesda). 2015 Oct 16;5(12):2759–73. doi: 10.1534/g3.115.023358 (PMC4683647; doi:10.1534/g3.115.023358)
Supplement: Supporting Information [file supp_g3.115.023358_TableS4.docx]

**Table S4.** Table S4. Genomic locations and PLAZA 3.0 gene families of PPR genes at the peach RF1 and RF2 loci (Donoso et al. 2015). The PPR gene family that contains known and suspected fertility restorers denoted with * in the notes column.

| **Gene** | **Linkage group** | **Start** | **End** | **PLAZA 3.0 gene family** | **PLAZA 3.0 orthology group** | **PLAZA 3.0 annotation (Proost et al 2015)** | **Notes** |
| --- | --- | --- | --- | --- | --- | --- | --- |
| ppa004557m | scaffold_2 | 1,188,729 | 1,190,329 | HOM03D000002 | ORTHO03D007411 | Pentatricopeptide repeat-containing protein At1g05600 | * |
| ppa003463m | scaffold_2 | 3,027,943 | 3,030,377 | HOM03D000001 | ORTHO03D016683 | Pentatricopeptide repeat-containing protein |  |
| ppa001444m | scaffold_2 | 3,894,642 | 3,897,125 | HOM03D000001 | ORTHO03D097346 | Pentatricopeptide repeat-containing protein |  |
| ppa021440m | scaffold_2 | 4,230,526 | 4,232,581 | HOM03D000002 | ORTHO03D014294 | Pentatricopeptide repeat-containing protein | * |
| ppa023365m | scaffold_6 | 5,768,164 | 5,769,950 | HOM03D000002 | ORTHO03D000298 | Pentatricopeptide repeat-containing protein At1g05670, mitochondrial | * |
| ppa015993m | scaffold_6 | 5,799,668 | 5,803,260 | HOM03D000774 | ORTHO03D008740 | Pentatricopeptide repeat-containing protein At1g07740, mitochondrial |  |
| ppa024218m | scaffold_6 | 5,906,881 | 5,908,413 | HOM03D000002 | ORTHO03D000298 | Pentatricopeptide repeat-containing protein At4g31850, chloroplastic | * |
| ppa025792m | scaffold_6 | 5,915,202 | 5,917,143 | HOM03D000002 | ORTHO03D000298 | Pentatricopeptide repeat-containing protein At4g31850, chloroplastic | * |
| ppa003571m | scaffold_6 | 6,024,610 | 6,028,036 | HOM03D000002 | ORTHO03D087015 | Putative pentatricopeptide repeat-containing protein At1g12700, mitochondrial | * |
| ppa026767m | scaffold_6 | 6,174,107 | 6,175,847 | HOM03D000002 | ORTHO03D023629 | Putative pentatricopeptide repeat-containing protein At1g12700, mitochondrial | * |
| ppa023798m | scaffold_6 | 6,182,493 | 6,184,532 | HOM03D000002 | ORTHO03D000298 | Pentatricopeptide repeat-containing protein | * |
| ppa019799m | scaffold_6 | 6,204,975 | 6,206,456 | HOM03D000002 | ORTHO03D000298 | Pentatricopeptide repeat-containing protein | * |
| ppa023651m | scaffold_6 | 6,234,407 | 6,235,708 | HOM03D000002 | ORTHO03D091095 | Putative pentatricopeptide repeat-containing protein At5g59900 | * |
| ppa023796m | scaffold_6 | 6,255,285 | 6,255,976 | HOM03D000002 | ORTHO03D297194 | Putative pentatricopeptide repeat-containing protein At1g12700, mitochondrial | * |
| ppa015333m | scaffold_6 | 6,260,699 | 6,262,145 | HOM03D000002 | ORTHO03D028908 | Pentatricopeptide repeat-containing protein At1g63130, mitochondrial | * |
| ppa017758m | scaffold_6 | 6,413,283 | 6,414,551 | HOM03D000002 | ORTHO03D121532 | Pentatricopeptide repeat-containing protein | * |
| ppa003306m | scaffold_6 | 6,474,068 | 6,475,828 | HOM03D000002 | ORTHO03D000298 | Putative pentatricopeptide repeat-containing protein At1g74580 | * |
| ppa019170m | scaffold_6 | 6,611,664 | 6,612,879 | HOM03D000002 | ORTHO03D008745 | Putative pentatricopeptide repeat-containing protein At1g12700, mitochondrial | * |
| ppa008492m | scaffold_6 | 6,616,986 | 6,618,084 | HOM03D000001 | ORTHO03D051096 | Pentatricopeptide repeat-containing protein |  |
| ppa025230m | scaffold_6 | 6,767,152 | 6,768,540 | HOM03D000001 | ORTHO03D009864 | Pentatricopeptide repeat-containing protein |  |
